# Potential Effects of Silymarin and Its Flavonolignan Components in Patients with *β*-Thalassemia Major: A Comprehensive Review in 2015

**DOI:** 10.1155/2016/3046373

**Published:** 2016-02-21

**Authors:** Hadi Darvishi Khezri, Ebrahim Salehifar, Mehrnoush Kosaryan, Aily Aliasgharian, Hossein Jalali, Arash Hadian Amree

**Affiliations:** ^1^Thalassemia Research Center, Mazandaran University of Medical Sciences, Sari, Iran; ^2^Faculty of Pharmacy, Thalassemia Research Center, Mazandaran University of Medical Sciences, Sari 48175-861, Iran

## Abstract

Major *β*-thalassemia (*β*-TM) is one of the most common inherited hemolytic types of anemia which is caused as a result of absent or reduced synthesis of *β*-globin chains of hemoglobin. This defect results in red blood cells lysis and chronic anemia that can be treated by multiple blood transfusions and iron chelation therapy. Without iron chelation therapy, iron overload will cause lots of complications in patients. Antioxidant components play an important role in the treatment of the disease. Silymarin is an antioxidant flavonoid isolated from* Silybum marianum* plant. In the present study, we reviewed clinical and experimental studies investigating the use of silymarin prior to September 1, 2015, using PubMed, ISI Web of Knowledge, Science Direct, Scopus, Ovid, and Cochrane Library databases and we evaluated the potential effects of silymarin on controlling the complications induced by iron overload in patients with *β*-TM. Based on the results of the present study, we can conclude that silymarin may be useful as an adjuvant for improving multiple organ dysfunctions.

## 1. Introduction

*β*-Thalassemia (*β*-TM) is a chronic hereditary disease with a high prevalence in the Mediterranean region, Middle East, Indian subcontinent, and South East Asia. So far, around 230 different mutations have been reported on *β*-globin gene worldwide [1, 2]. The ineffective red blood cell (RBC) synthesis in these patients due to unbalanced hemoglobin chains production cause increased RBCs turnover and anemia that can be ameliorated by blood transfusions [3]. Although recurrent blood transfusion could be an effective treatment and reduces disease-specific morbidity and mortality, it is a comprehensive source of iron overload that can have several side effects [4, 5]. Despite iron chelation therapy, chronic transfusion therapy often leads to massive iron overload in liver, heart, brain, and endocrine organs and subsequent organ dysfunction that ultimately results in death [3, 6, 7]. Iron overload may also occur in patients who do not receive multiple blood transfusions due to the absorption from the gut [8]. Oxidative stress, inflammation, hepatic involvements, osteoporosis, and cardiac and renal insufficiency are major causes of iron overload related morbidity in patients with *β*-TM [9].

In the past decade, the protective activities of various herbal flavonoids have been investigated.* Silybum marianum* (St. Mary's thistle, milk thistle; Asteraceae/Flavonolignan) was widely used in traditional European medicine for 2000 years especially for the treatment of the liver, spleen, and gallbladder disorders [10]. The seeds are the active part of the plant and the main flavonoid of them is called silymarin which consists of a mixture of four flavonolignans (70–80%): silibinin (silybin) (50%), silychristin (20%), silydianin (10%), and isosilybin (5%) [11]. Silybin is the most important biologically active component of silymarin complex [12].

In the present study, we investigated the protective properties of silymarin and its constituents in *β*-TM patients to consider the clinical applications of this herbal extract for protection against iron-induced organs damage.

## 2. Methods

We searched the English literature in PubMed, ISI Web of Knowledge, Science Direct, Scopus, Ovid, and Cochrane Library databases to find studies published from January 2000 to September 2015. The titles of the searches were appropriate MESH headings including “Milk thistle”, “silymarin”, “silybum”, “silibinin”, “silybin”, “silydianin”, “silychristin”, “herbs”, “medicinal plant”, “natural product”, “herbal medicine”, “plant medicine”, “phytomedicine”, and “thalassemia”. Moreover, in addition to the electronic searches, manual searches of reference lists used in all of the retrieved review articles and primary studies were carried out to identify other studies that were not found in the electronic searches. The literature was searched by two authors independently. The inclusion criteria of the papers were as follows: (1) the studies on antioxidant effects, iron chelating, liver protective, anti-inflammatory, immunomodulatory, and antiosteoporotic activities, and cardiac and renal protective effects that were conducted on animals and humans; (2) plant extracts or compounds isolated from plant. The exclusion criteria consisted of (1) the studies that were about an herbal formula and (2) the articles that were not written in English or translated to English. Two researchers independently read the full texts and extracted the following contents: publication data; study design; sample size; patient characteristics; treatment protocol; and outcome measures.

**Figure 1:**
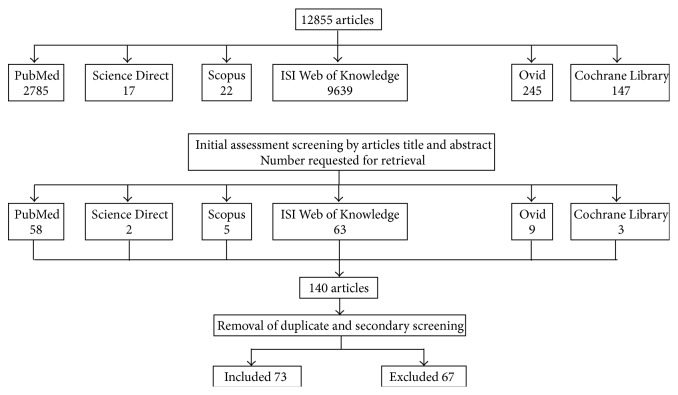
Literature search and retrieval flowchart.

The search strategy generated 12855 titles and abstracts. After initial screening and evaluation, 12715 articles were rejected and 140 articles were founded to be potentially eligible for the review. These articles were retrieved for full text review. Removing duplicates and using secondary screening resulted in 73 articles to be included for the review (Figure 1).

## 3. Results

### 3.1. Antioxidant Effects

Iron toxicity in *β*-TM is the main cause of oxidative stress. Oxidative stress, associated with the formation of reactive oxygen species (ROS), plays an important role in the development of inflammation, decreased level of plasma antioxidants, depletion of erythrocyte glutathione (GSH), increased lipid peroxidation of RBC membranes, and immunosuppression in these patients [13, 14]. Several studies have shown that silymarin modulates imbalance between cell survival and apoptosis through interference with the expressions of the cell cycle regulators and proteins involved in apoptosis [15]. Silymarin protects cells from ROS damages by increasing endogenous antioxidant enzymes such as glutathione peroxidase (GPx) and superoxide dismutase (SOD). Moreover, it also inhibits the activation of NF-*κ*B [16, 17].

Alidoost et al. surveyed intracellular GSH and proliferative response of peripheral blood mononuclear cells (PBMC) before and after 72-hour incubation of PBMC with various concentrations of silymarin (0, 5, 10, or 20 *μ*g/mL) in 28 patients with *β*-TM and 28 healthy age-matched individuals [14]. Results of that study showed a significant restoration of GSH and its normalization in *β*-TM cells following treatment with silymarin. GSH is a primary intracellular antioxidant and plays an essential role in several functions in T cells [14]. Considerably, low levels of GSH and depressed proliferative response of PBMC in *β*-TM may be responsible for the cell mediated immune abnormalities in iron overload conditions [14]. These data indicate the benefit of using silymarin to normalize immune dysfunction via antioxidant and immunostimulatory activities in *β*-thalassemia major. Moreover, Jeong et al. found that silibinin can successfully prompt apoptosis and as a result it leads to human glioma tumor cells death through calpain-dependent mechanism involving protein kinase C (PKC), ROS, and apoptosis-inducing factor (AIF) nuclear translocation [18].

### 3.2. Iron Chelation Effects

For the first time, Borsari et al. introduced silybin as a new iron-chelating agent [19] and since then the in vitro studies have showed that silybin has a high affinity for Fe (III) at acidic pH and makes an iron-silybin complex [12, 20]. Consequently, some clinical trials have reported that silymarin and silybin may act as iron-chelating agents in patients with *β*-TM [11, 21, 22]. These studies argued that treatment with silymarin and silybin leads to reduction in the body iron stores and decrease of serum ferritin level.

Navidi-Shishaone et al. investigated the effect of desferrioxamine (DFO) and silymarin combination therapy against kidney and heart iron deposition in an iron overload rat model. The iron overload condition was performed by iron dextran (100 mg/kg/day) every other day. Administration of iron dextran was stopped after 2 weeks and the animals were treated daily with combination of silymarin (200 mg/kg/day, intraperitoneally) and DFO (50 mg/kg/day, intraperitoneally). The results displayed that although coadministration of silymarin and DFO may be potentially considered as an iron chelator, combination of these two agents does not reduce the intensity of iron deposition in the kidney, liver, and heart [23]. In a clinical trial by Gharagozloo et al., 59 patients with *β*-TM were randomized into two groups. First group received silymarin plus DFO while DFO with placebo was administered for the second group for 3 months (Table 1 shown). Findings of the mentioned study indicated that the combination therapy was well tolerated and more effective than DFO alone in reducing serum ferritin level. However, no significant differences were detected between silymarin and placebo groups in serum ferritin level after 1.5 and 3 months of treatment. In that study, it was also argued that the observed slight changes in ferritin level between two groups probably may be because of small sample size. That was the first report showing the beneficial effects of silymarin in *β*-TM patients [11]. In another study, patients were treated with the combination of DFO and silymarin (420 mg/day) or DFO plus placebo (49//versus 48//) for 9 months. Serum iron and total iron binding capacity (TIBC) levels were significantly reduced after silymarin therapy. Moreover, serum ferritin levels strongly decreased in silymarin group in comparison to placebo group (Table 1). That shows potential effectiveness of silymarin alone as an iron-chelating agent in reducing body iron load in *β*-TM [21]. Balouchi et al. stated that although 69.23% of patients have a little drop in the serum ferritin level in combination therapy group (DFO plus 420 mg/day silymarin), reduction of serum ferritin level was not significant after 6 months (Table 1 shown). Besides, they mentioned that silymarin has iron chelator effects and the little sample size was the reason why the nonsignificant decrease in serum ferritin level was observed [24]. Recently, Hagag et al. investigated therapeutic effects of silymarin plus deferiprone (DFP) in 80 *β*-TM patients with > 1000 ng/mL serum ferritin level [22]. They indicated that, after treatment, the serum ferritin and iron levels were dramatically decreased. In addition, higher TIBC was also observed after combination therapy (Table 1). These findings are supported by results of their previous study with deferasirox (DFX) and silymarin combination therapy [25]. Moreover, after combination therapy, serum iron level was significantly decreased from 248.85 ± 38.2 to 137.4 ± 31.1 ng/dL (*P* = 0.001). Based on the results of these two studies, it can be concluded that the iron chelator effects of silymarin are related to its ability of Fe (III) binding. Similarly, Bares et al. evaluated that administration of oral silybin for 12 weeks decreases body iron stores in patients with chronic hepatitis C [26]. It seems that silymarin could potentially have an iron-chelating effect via strong antioxidant activity and reducing nonhaem iron [20]. More studies are required to clarify the role of silymarin in the decrease of iron overload in clinical condition.

### 3.3. Hepatic Protection

Iron overload can affect various tissues including liver in patients with *β*-TM. Liver injury and chronic hepatitis (B and C) are serious medical problems in the transfusion dependent *β*-TM patients. The high incidence of hepatitis C was observed in *β*-TM patients after a screening during a fourteen-year study (at two 7-year intervals; 1996–2002 and 2003–2009). Of the 395 patients, 109 (27.5%) were anti-HCV positive, and 21 (19.2%) out of these 109 cases were exposed after 1996. The incidence rate of HCV was 4.2/1000 person-years during that time. The incidence rates of HCV in the first and second seven-year periods were 6.2/1000 and 1.3/1000 person-years, respectively [27].

Silymarin has been extensively used as a hepatoprotective agent in Asia and Europe [18]. Although it has been widely used in the treatment of liver disease, few clinical trials have been conducted about its effects on patients. In animal models, silymarin was shown to protect the patients against liver injury induced by toxins [28]. Based on findings of cellular morphological changes in rat using light microscope, the protective roles of silymarin plus DFO on iron overload-induced hepatotoxicity were introduced by Najafzadeh et al. [29]. The mentioned study showed antihepatotoxic effects of silymarin, improvement of liver function, and decrease of total protein and total albumin in animal models. Studies of silymarin at high concentrations in the HCV replicon system also show an effect on HCV core and NS5A gene expression [30, 31]. In these studies, an anticarcinogenic role of silymarin was argued to be the main mechanism of its actions [10]. The results of these studies demonstrate that silymarin has antiviral and hepatoprotective effects and it may be useful in the treatment of *β*-TM patients with hepatitis as a complementary approach.

### 3.4. Immunomodulatory and Anti-Inflammatory Effects

There are several immune abnormalities in transfusions dependent *β*-TM patients such as increased number and enhanced activities of suppressor T-cells (CD8), reduced proliferative capacity of helper T-cells (CD4), and decreased CD4/CD8 ratios [32–34]. Moreover, iron overload in *β*-TM patients reduces the proliferative activities of T cells [35, 36]. Iron overload and continuous immune stimulation are the key causes of suppressed cell mediated immunity in these patients [36, 37].

Immunomodulatory and anti-inflammatory effects of silibinin have been shown in some studies. Silibinin inhibits tumor necrosis factor alpha (TNF-*α*) production [38, 39]. Silibinin suppresses the growth of HMC-1 cells and it also decreases expression of proinflammatory cytokines through inhibition of NF-*κ*B signaling pathway in HMC-1 human mast cells (such as TNF-*α*, IL-6, and IL-8) [40]. An in vitro study showed that silymarin has anti-inflammatory and immunomodulatory effects through inhibition of NF-*κ*B [41, 42]. Wilasrusmee et al. reported that in vitro treatment of PBMC with silymarin leads to restoration of the thiol status and increases T cell proliferation and activation, and it enhances interferon gamma (IFN*γ*), interleukin- (IL-) 4, and IL-10 secretions via stimulating of the lymphocytes in a dose dependent pattern [43]. So, they recommended the silymarin as a possible effective immunomodulatory herbal medication in the management of *β*-TM patients because of its antioxidant, cytoprotective, and iron-chelating activities [3].

Gharagozloo et al., in a 12-week randomized clinical trial, examined the immunomodulatory effects of silymarin. Twenty-five patients received DFO (40 mg/kg/day) and 420 mg of silymarin daily as a combination therapy while five cases took only silymarin. The serum levels of neopterin and TNF-*α* were dramatically diminished in two groups. Neopterin is created by monocytes and macrophages upon stimulation with IFN-*γ*. Measurement of neopterin blood concentrations provides important information about the triggering of cellular immune activation in humans under the control of T helper cells type 1. This analysis allows researchers to evaluate the extent of oxidative stress stimulated by the immune system [3]. Moreover, increased production of IFN *γ* and IL-4 was observed in activated T cells following silymarin therapy in both groups. Based on these results, Gharagozloo et al. concluded that silymarin may stimulate cell-mediated immune response via a direct effect on cytokine-producing mononuclear cells in *β*-TM. Besides, there were no evidences of lymphocyte subsets percentage, concentration of serum immunoglobulins, complement levels, and T cell proliferation between intervention groups [3].

Researchers proposed that probable iron-chelating and antioxidant activities of silymarin can be considered as an important mechanism of its immune-stimulatory effect. They also stated that silymarin has strong dose dependent immunomodulatory effects. Interestingly, it shows an immune-stimulatory activity at low doses (40/mg/kg/day) and immunosuppressive effects at high doses [3, 44]. Schümann et al. indicated that silymarin suppresses T cell-dependent liver injury and inhibits intrahepatic expression of TNF-*α*, IFN-*γ*, IL-4, IL-2, and inducible nitric oxide synthase (iNOS) in in vitro condition [45]. It has been shown that silymarin strongly disturbs the activation of NF-*κ*B and mTOR in activated T lymphocytes with inhibition of IL-2 and IFN-*γ* production and cell proliferation [46, 47].

A clinical study investigated the immunomodulatory effect of silymarin (420 mg/day) by measuring the serum levels of TGF-*β*, IL-10, IL-17, and IL-23 in patients with *β*-TM in comparison to healthy controls. The results showed a significant higher concentration of TGF-*β* and IL-23 in the patient group than the controls. Among cytokines, only a significant reduction in serum IL-10 levels was found due to silymarin administration. In patients treated with silymarin, a fall in serum TGF-*β* (38%), IL-10 (84.6%), IL-17 (61.5%), and IL-23 (61.5%) levels was noted. This data propose that the immune abnormality, inflammation, and immunosuppression caused by iron overload in *β*-TM patients could be modulated by silymarin [24]. These results suggest that silymarin and its compounds could ameliorate immune abnormalities and inflammation in *β*-TM patients.

### 3.5. Osteoprotective Effects

Osteoporosis is a common bone-related metabolic disease characterized by low bone density and increased bone fragility and fractures [48]. Patients with *β*-TM are susceptible to osteopenia and osteoporosis. The mechanism of osteoporosis in these patients is multifactorial. Iron deposition in endocrine organs following multiple transfusion leads to impaired growth hormone secretion, hypothyroidism, hypoparathyroidism, lack of gonadal steroids, and vitamin D deficiency which contribute to the defect in achieving an acceptable bone density [49]. Recently, estrogenic and osteoprotective effects of silymarin were studied in animal models. Some studies found that silibinin has a potential to increase osteoblastogenesis and inhibit osteoclast formation by attenuating the downstream signaling cascades associated with receptor activator of NF-*κ*B ligand or TNF-*α* in murine preosteoblastic cell. In addition, silibinin might act as bone morphogenetic protein (BMP) modulator, osteoprotective, and inhibitor of osteoclastic bone resorption. BMPs are a group of growth factors known as cytokines and metabologens that induce the development of bone and cartilage. BMPs are now considered to constitute a group of pivotal morphogenetic signals and orchestrating tissue architecture throughout the body [50, 51]. Silymarin therapy may also heighten collagen secretion, osteocalcin transcription, and BMP expression [52]. Seidlová-Wuttke et al. demonstrated that silymarin is a selective estrogen receptor modulator (SERM) on the ER*β*-subtype of the estrogen receptor and it can be considered as pure ER*β*-specific ligands [53]. ER*α* and ER*β* are the classical estrogen receptors that engage in the regulation of many complex physiological processes in humans. Modulation of these receptors is now considered for the treatment and prevention of osteoporosis [54]. Although several studies have established the various roles of silymarin in both in vitro and in vivo models, the effect of silymarin and its flavonolignan components as an osteoprotective agent in clinical practice is required to be investigated.

### 3.6. Cardiac Protective Effects

Cardiac complications such as cardiomyopathy and heart failure secondary to iron overload are still the main cause of mortality in *β*-TM [55]. It has been shown that around 70% of deaths are related to this complication [56]. Recently, some randomized clinical trials were carried out on these patients regarding cardiac protective property of silymarin [11, 19, 21]. It seems that decrease of oxidative stress markers such as ROS inside the heart cells is caused by strong antioxidant properties of silymarin and their cytoprotective and anti-inflammatory effects could be responsible for cardioprotective effects [57, 58]. Moreover, silymarin protects cardiac myocytes via decrease of lactate dehydrogenase (LDH) and malondialdehyde (MDA) [59]. However, the cardiac protective effects of silymarin are not clear in clinical researches.

### 3.7. Renal Protective Effects

Renal dysfunction as result of tissue iron deposition is one of the main problems in patients with *β*-TM [60]. Currently, a few researches showed that silymarin can protect kidney against induced iron toxicity in *β*-TM and diabetics patients and also after chemotherapy in cancer patients [61–65]. Silymarin significantly can reduce kidney iron deposition in rat model and it has nephroprotective properties in acute iron overload animal models. Fallahzadeh et al. stated that silymarin can be considered as a new addition to the antidiabetic nephropathy armamentarium [66]. In this clinical trial, urinary albumin-creatinine ratio (UACR), urinary levels of TNF-*α*, and urinary and serum levels of MDA were significantly reduced in the intervention group. Silymarin could improve diabetic nephropathy at 140 mg doses 3 times a day for 3 months in type 2 diabetes patients with macroalbuminuria. For better understanding of the renal protective effects of silymarin, more studies are recommended to carry out, especially, evaluation of administration of silymarin in combination with standard iron chelation.

## 4. Conclusion

According to the current review, it seems that silymarin plus standard iron chelation may have a better result to protect organ induced iron overload. Silymarin which is a safe and well-tolerated adjuvant is introduced as a drug without adverse effects in many clinical studies. We recommend that more well-designed randomized clinical trials are required considering silymarin pharmacokinetic behavior in order to generate strong evidence about improvement of iron overload complications among patients with *β*-TM.

## Figures and Tables

**Table 1:** Summary of carried-out randomized clinical trials of silymarin in patients with *β*-TM.

Study author (reference)	Methods					Ferritin (ng/mL)				
	Groups		Silymarin dose	Study design	Period of study (week)	Intervention group		Control group		*P* value between groups
	Intervention (*N*)	Control (*N*)				Baseline	End	Baseline	End	
Gharagozloo et al., 2009 [11]	DFO plus silymarin (*n* = 29)	DFO plus placebo (*n* = 30)	40 mg/kg/day	Parallel	12	4285.4 ± 2181.3	3548.3 ± 2012.8	3772.7 ± 1806.6	3727.8 ± 2025.0	NS

Moayedi et al., 2013 [21]	DFO plus silymarin (*n* = 49)	DFO plus placebo (*n* = 48)	40 mg/kg/day	Parallel	36	3028.8 ± 2002.6	1972.2 ± 1250.6	1780.19 ± 1089.5	2213.8 ± 1375.1	0.01

Gharagozloo et al., 2013 [3]	silymarin (*n* = 5)	DFO plus silymarin (*n* = 25)	40 mg/kg/day	Parallel	12	NM	NM	NM	NM	—

Balouchi et al., 2014 [24]	DFO plus silymarin (*n* = 13)	DFO plus placebo (*n* = 9)	40 mg/kg/day	Parallel	24	2292.20 ± 1382.26	1935.70 ± 1649.35	1701.56 ± 773.51	1794.67 ± 870.65	NS

Hagag et al., 2013 [25]	DFX plus silymarin (*n* = 20)	DFX plus placebo (*n* = 20)	40 mg/kg/day	Parallel	24	3253.7 + 707.1	1067.2 + 297.9	3049.2 + 527.7	1795.3 + 551.6	0.001

Hagag et al., 2015 [22]	DFP plus silymarin (*n* = 40)	DFP plus placebo (*n* = 40)	40 mg/kg/day	Parallel	36	1901 + 563.38	989.5 + 178.57	1885.2 + 510.54	1260 + 212.26	<0.001

NS: nonsignificant.

NM: nonmeasured.
